# CX3CR1–Fractalkine Dysregulation Affects Retinal GFAP Expression, Inflammatory Gene Induction, and LPS Response in a Mouse Model of Hypoxic Retinopathy

**DOI:** 10.3390/ijms26031131

**Published:** 2025-01-28

**Authors:** Colin Rorex, Sandra M. Cardona, Kaira A. Church, Derek Rodriguez, Difernando Vanegas, Reina A. Saldivar, Amira El-Sheikh, Yufeng Wang, Stefka Gyoneva, Anne C. Cotleur, Astrid E. Cardona

**Affiliations:** 1 Department of Molecular Microbiology and Immunology, The University of Texas at San Antonio, San Antonio, TX 78249, USA; 2 South Texas Center for Emerging Infectious Diseases, The University of Texas at San Antonio, San Antonio, TX 78249, USA; 3 Natural and Physical Science, Northwest Vista College, San Antonio, TX 78251, USA; 4 Cerevel Therapeutics, Cambridge, MA 02142, USA; 5 Biogen, Cambridge, MA 02142, USA

**Keywords:** diabetic retinopathy, astrocytes, microglia, Müller glia, hypoxia, inflammation, fibrinogen

## Abstract

Diabetic retinopathy (DR) causes vision loss due to sustained inflammation and vascular damage. The vascular damage is evident by fibrinogen leakage, angiogenesis, and hypoxia. Neuronal regulation of microglia via the CX3CL1 (Fractalkine or FKN)-CX3CR1 pathway plays a significant role in retinal pathology. Defects in FKN or CX3CR1 exacerbate inflammation, vascular damage, and vision impairment. However, the contribution of hypoxic astrocytes to the pathological process of DR is unclear. A hypoxic model (7 days of systemic 7.5% O_2_) was utilized to induce retinal damage in adult mice in the absence of systemic inflammatory signals. This model induced vascular and microglial responses similar to 10 weeks of STZ-induced hyperglycemia. The goal of this study is to characterize retinal damage in WT and mice with defects in the FKN-CX3CR1 signaling axis and hence assess the impact of the microglial inflammatory responses to hypoxic retinopathy. Tissues were analyzed by immunostaining, RNA sequencing, and cytokine quantification. We found that CX3CR1 deficiency in hypoxic animals induced reactive astrogliosis and that Müller glial responses to hypoxia and systemic inflammation were dependent on FKN signaling. Exacerbated microglial reactivity to hypoxic conditions significantly altered the expression of HIF transcripts. Microglial dysregulation was found to reduce the anti-inflammatory response to hypoxic conditions, downregulate hypoxia-responsive gene expression, and restrained LPS-induced inflammatory responses. We found that microglia dysregulation alters the hypoxic response by inhibiting the upregulation of HIF2α/3α, increasing CD31 immunoreactivity, and altering the expression of ECM-associated transcripts such as type I, III, and XVIII collagens to hypoxic conditions.

## 1. Introduction

Fractalkine (FKN), a chemokine expressed on neuronal membranes, plays a crucial role in the communication between neurons, microglia, and astrocytes, particularly in the context of diabetic retinopathy (DR). FKN signals through its unique receptor CX3CR1, which is predominantly expressed on microglia, the resident immune cells of the central nervous system (CNS) [1]. In the retina, microglia are integral to the neurovascular unit, interacting closely with neurons and essential to immunosurveillance [1]. During early diabetes, microglial activation is characterized by recruitment to vascular lesions, leading to capillary constriction, reducing blood flow, maintaining vascular integrity, and aiding in wound healing [2,3]. This microglial activation is modulated by FKN/CX3CR1 signaling, which transduces inhibitory signals to ameliorate microglial activation and proinflammatory cytokine release [4]. Dysregulation of this signaling pathway exacerbates inflammatory responses, as seen in CX3CR1-deficient mice, which exhibit increased microglial numbers, elevated IL-1β levels, and enhanced neuronal damage in the diabetic retina [5]. Astrocytes, another critical component of the neurovascular unit, also respond to diabetic conditions by producing inflammatory cytokines like IL-1β, further potentiating microglial activation and contributing to vascular leakage and neuronal damage [5,6]. Additionally, FKN has been shown to deactivate microglia by inhibiting the NF-κB pathway and activating the Nrf2 pathway, thereby reducing the production of reactive oxygen species (ROS) and inflammation-related cytokines, hence protecting the retina from diabetic insults [7].

Astrocytes, along with Müller cells, are integral to maintaining retinal homeostasis by providing physical support to neurons and interacting with all retinal vascular layers to maintain the blood–retinal barrier (BRB) and regulate local blood flow under physiological conditions [8,9]. In DR, astrocytes exhibit reactive gliosis in response to hyperglycemia-induced stress, which can lead to both neuroprotective and cytotoxic effects [8]. Specifically, under hypoxic conditions, which are common in DR, astrocytes and Müller cells significantly upregulate the production of vascular endothelial growth factor (VEGF). This upregulation is crucial due to VEGF’s role as a potent mitogen for endothelial cells and plays a critical role in pathological angiogenesis and increased vascular permeability, contributing to the progression of DR [10]. Furthermore, astrocytes contribute to the accumulation of metabolic by-products in the capillary walls, exacerbating microvascular damage and occlusion, which are hallmarks of DR [11]. Neurotoxic astrocyte responses are driven by a combination of disease-induced tissue stress and inflammatory mediators produced by reactive microglia [12], while neuroprotective responses are driven by ischemic neuronal prokineticin 2 signaling [13,14]. However, hypoxic astrocyte responses do not appear essential for normal retinal development [15]. Systemic hypoxia plays a significant role in the pathogenesis of various retinal diseases, particularly through mechanisms involving vascular changes [16] and inflammation [17]. The crosstalk between astrocytes and microglia to the mechanism of DR pathology is not well understood as both chronic inflammation and ischemia are present in the diabetic retina.

To investigate the contributions of astrocytes to DR pathology, we utilized a model of hypoxia-induced retinopathy where pathology is initiated by oxygen insufficiency. We combined hypoxic retinopathy with mouse models of defective FKN-CX3CR1 signaling, which alter the inflammatory responses in CNS tissues, creating a tissue environment with exacerbated inflammatory responses [18]. Here, we hypothesize that dysregulation of FKN-CX3CR1 signaling will attenuate the anti-inflammatory hypoxic astrocyte response with increased astrocyte and Müller glia reactivity, inhibition of angiogenesis regulation, and increased expression of inflammatory mediators.

Our findings, as published in similar studies [19,20], reveal that a 7-day hypoxia model in WT mice induces microglial reactivity and vascular damage, suggesting that dysregulated microglia not only impact the apoptotic response to hypoxia but also alter early angiogenic tissue responses. Additionally, retinal inflammation induced via microglial dysregulation was found to downregulate hypoxia-responsive gene expression and prevent the inhibition of LPS-induced inflammatory responses. These results suggest a complex interaction between inflammation, hypoxia, and vascular damage during retinopathy and limiting inflammatory responses in CNS diseases with hypoxic components may enhance neuroprotection and promote anti-inflammatory astrocyte activity. Collectively, these findings underscore the importance of FKN-mediated microglia and astrocyte communication in mitigating the progression of DR and highlighting potential therapeutic avenues for preserving retinal health.

## 2. Results

### 2.1. Microglia Reactivity, Reduced GFAP Expression, and Vascular Damage Are Observed in Both Models of Hypoxia- and STZ-Induced Retinopathy

**Figure 1:**
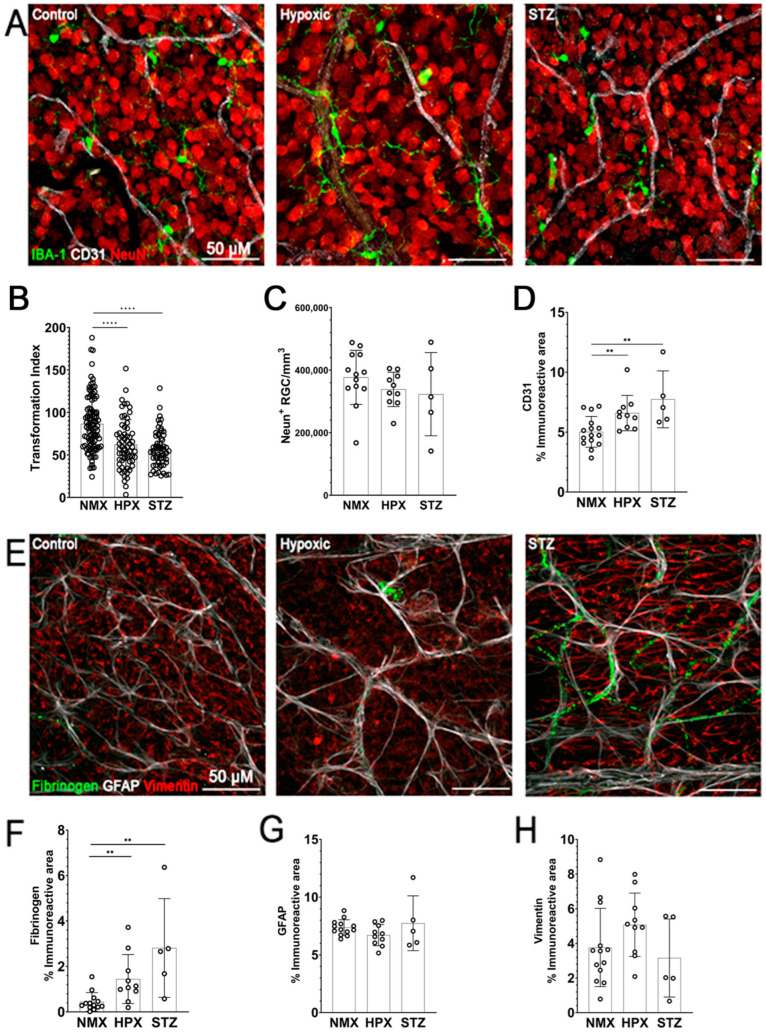
Hypoxia induces a similar phenotype of retinal pathology compared to 10-week STZ-induced hyperglycemia in C57BL/6J mice. (**A**) Representative 40× magnification confocal images of mouse retinas stained with microglia marker IBA-1 (green), vascular marker CD31 (white), and retinal ganglion cell marker NeuN (red). (**B**) Microglia reactivity is quantified by the transformation index and the density of IBA-1+ cells. (**C**) Quantification of RGC density. (**D**) Quantification of retinal endothelial cells by CD31+ immunoreactivity. (**E**) Representative 40× magnification confocal images of mouse retinas stained with astrocyte marker GFAP (white), Müller glia marker vimentin (red), and blood protein fibrinogen (green). (**F**–**H**) Quantification of immunoreactive areas for fibrinogen, GFAP, and vimentin, respectively. Data were analyzed for statistical significance across normoxic (NMX), hypoxic (HPX), and diabetic (STZ) groups. Data show mean ± SD, n = 10, WT NMX, n = 10 WT HPX, and n = 5 STZ. Dots indicate individual microglia (**B**) or averages of IR area for corresponding staining per individual mice (**C**,**D**,**F**,**H**). Scale bar measures 50 μm. ** *p* < 0.01, **** *p* < 0.001 using Student’s *t*-test with Welch’s correction.

We hypothesized that a model of hypoxia-induced retinopathy could produce a phenotype of retinal damage similar to our models of STZ-induced DR at a 10-week time point. To evaluate the impact of hypoxia on glial cell reactivity and vascular pathology, retinal samples from WT mice exposed to 7 days of hypoxic conditions (HPX) were compared against 10-week STZ-induced diabetic (STZ) and normoxic control (NMX) tissues. Immunostaining of retinal leaflets revealed microglia (IBA-1, green), retinal ganglion cells (NeuN, red), and endothelial cells (CD31, white) (Figure 1A). Significant changes in microglia morphology were detected in the HPX (62.29 ± 29.18, *p* < 0.0001) and STZ (57.05 ± 21.25, *p* < 0.0001) retinas in comparison to the NMX controls (86.48 ± 32.46) (Figure 1B). These findings imply that both hypoxia- and STZ-induced hyperglycemia elicited similar levels of microglia activation. Examination of neurodegeneration unveiled that the RGC densities remain consistent when comparing the NMX (376,852 ± 86,395 cells/mm^3^) with STZ (322,974 ± 132,961 cells/mm^3^, *p* = 0.9024) and HPX retinas (338,411 ± 54,994 cells/mm^3^, *p* = 0.2340) (Figure 1C). These results suggest that neither a 10-week duration of STZ-induced diabetes nor 7-day period of hypoxia proved adequate to induce neurodegeneration at these time points. Subsequently, we tested for evidence of vascular abnormalities based on alterations in the CD31 immunoreactive area and fibrinogen accumulation in retinal tissues. In contrast to NXM (5.025 ± 1.283%), the STZ (7.738 ± 2.377%, *p* = 0.0040) and HPX tissues exhibited a significantly higher CD31 immunoreactive area (6.592 ± 1.477%, *p* = 0.0098) (Figure 1D). We also evaluated the response of astrocytes (GFAP, white), Müller glia (vimentin, red), and fibrinogen (green) to hypoxia (Figure 1E). There was an indication of vascular leakage with fibrinogen accumulation significantly elevated in the HPX (1.447 ± 1.078% vs. control 0.4397 ± 0.4116%, *p* = 0.0054) and STZ (2.813 ± 2.174%, vs. control *p* = 0.012) tissues (Figure 1F). These findings indicate that hypoxia can induce vascular leakage and increase CD31 expression similar to STZ-induced DR. Next, we examined the macroglia response. In comparison to the NMX tissues (7.364 ± 0.6771%), neither the HPX (6.708 ± 0.9216%) nor STZ tissues (7.738 ± 2.377%) showed a statistically significant change in GFAP immunoreactivity (Figure 1G). When evaluating the changes in the vimentin immunoreactive area (Figure 1H), there was no significant change between the NMX (3.765 ± 2.259%), HPX (5.074 ± 1.829%), and STZ tissues (3.156 ± 2.253%). These results suggest that there is neither an astrocyte nor Müller glia response to hypoxic or STZ treatment alone. Overall, these findings suggest that hypoxia triggers a similar phenotype of reactive microglia and vascular leakage as STZ-induced diabetes.

### 2.2. Hypoxia Induces Reactive Microgliosis, but Astrocyte and Müller Glial Responses to Hypoxia and Systemic Inflammation Are Dependent on Specific Defects in FKN-CX3CR1 Signaling

**Figure 2:**
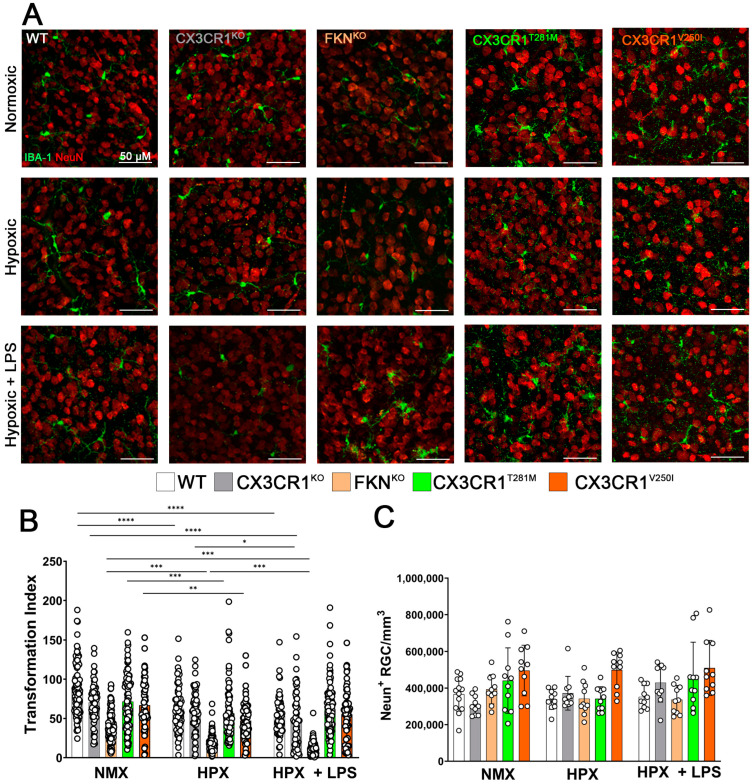
Hypoxia induces reactive microglia activation in models of FKN-CX3CR1 signaling dysregulation. (**A**) Representative 40× magnification confocal images of mouse retinas stained with microglia marker IBA-1 (green) and retinal ganglion cell marker NeuN (red). (**B**) Quantification of microglia reactivity by transformation index and RGC density, respectively. (**C**) Quantification of RGC cell density. Data show mean ± SD, n = 10 mice per group, dots indicate (**B**) individual microglia or (**C**) averages for individual mice. Scale bar measures 50 μm. * *p* < 0.05, ** *p* < 0.01, *** *p* < 0.001, **** *p* < 0.0001 using one-way ANOVA with multiple comparisons and Tukey’s correction.

In our previous work, we reported that disrupting the FKN-CX3CR1 signaling axis increases microglia activation and inflammation and worsens retinal pathology in a diabetic mouse model. Hence, we aimed to examine how genetic models with defective FKN-CX3CR1 signaling would impact astrocyte responses under hypoxia-induced retinopathy. Groups of WT, FKN^KO^, CX3CR1^KO^, CX3CR1^V250I^, and CX3CR1^T281M^ mice were subjected to hypoxic conditions alone or in conjunction with LPS-induced systemic endotoxemia (HPX + LPS). Initially, we assessed if models of microglia dysregulation influenced microglia reactivity (IBA-1, green) and RGC density (NeuN, red) (Figure 2A). We observed a notable decrease in the transformation index in WT retinas in response to hypoxic conditions, which was not worsened by the two-hit model we employed (NMX 86.48 ± 32.46 vs. HPX 62.29 ± 29.18 *p* < 0.0001, NMX vs. HPX + LPS 53.49 ± 24.60, *p* < 0.0001). Both CX3CR1^KO^ (NMX 70.51 ± 25.44 vs. HPX 58.69 ± 26.39 *p* = 0.0651, NMX vs. HPX + LPS 64.01 ± 34.65, *p* < 0.0001, and HPX vs. HPX+ LPS, *p* = 0.0468) and FKN^KO^ retinas (NMX 39.23 ± 18.88 vs. HPX 18.58 ± 9.221 *p* < 0.0001, NMX vs. HPX + LPS 11.70 ± 7.533, *p* < 0.0001, and HPX vs. HPX + LPS *p* < 0.0001) showed an exacerbation in microglia reactivity with the addition of LPS. However, both CX3CR1^T281M^ (NMX 71.493 ± 34.70 vs. HPX 56.60 ± 33.32 *p* = 0.0061, NMX vs. HPX+LPS 61.31 ± 30.25, *p* = 0.0659), and HPX vs. HPX + LPS *p* = 0.5474) and CX3CR1^V250I^ tissues demonstrated that hypoxia alone led to a significant increase in microglia reactivity (TI values in NMX 66.88 ± 31.61 vs. HPX 47.36 ± 24.44, *p* = 0.0011, NMX vs. HPX + LPS 55.32 ± 31.92, *p* = 0.0084, HPX vs. HPX + LPS *p* = 0.3044) (Figure 2B). These findings indicate that disruptions in the CX3CR1-FKN signaling axis may affect the microglia response to hypoxia. Despite evidence of hypoxia-induced microglia reactivity, there was no evidence of neurodegeneration in the WT, CX3CR1^KO^, FKN^KO^, CX3CR1^T281M,^ or CX3CR1^V250I^ retinas (Figure 2C). These results show that while hypoxia in the two-hit model induces reactive microglia, no neurodegeneration was observed in any of the models of microglia dysregulation.

**Figure 3:**
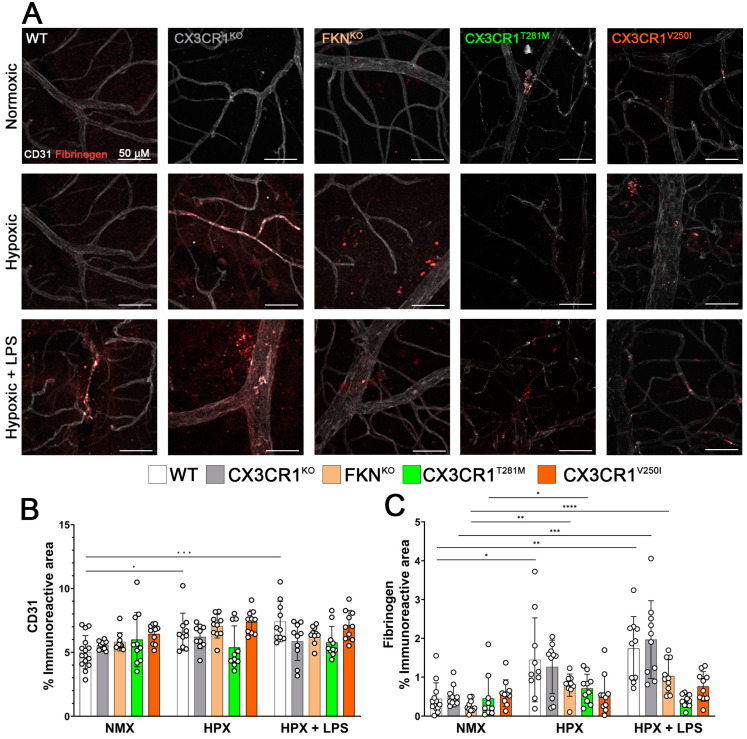
Vascular leakage is induced by hypoxia in WT retinas but addition of LPS-induced systemic inflammation is required for vascular leakage in CX3CR1^KO^ and FKN^KO^ retinas. (**A**) Representative 40× magnification confocal images of mouse retinas stained with endothelial cell marker CD31 (white) and fibrinogen (red) (**B**,**C**). Quantification of CD31 and fibrinogen immunoreactive areas, respectively. Data show mean ± SD, n = 10 mice per group, dots indicate average for individual mice. Scale bar measures 50 μm. * *p* < 0.05, ** *p* < 0.01 *** *p* < 0.001, **** *p* < 0.0001 using one-way ANOVA with multiple comparisons and Tukey’s correction.

We next sought to test for evidence of vascular pathology by staining for endothelial marker CD31 (white) and clotting factor fibrinogen (red) (Figure 3A). We found evidence of increased CD31 immunoreactivity in the WT tissues in response to hypoxic conditions but not exacerbated by the addition of LPS-induced systemic inflammation (NMX 5.025 ± 1.283% vs. HPX 6.591 ± 1.477% *p* = 0.0296, NMX vs. HPX + LPS 7.398 ± 1.582% *p* = 0.0008, NMX vs. HPX + LPS *p* = 0.4254). There was no change in CD31 immunoreactivity in response to hypoxic conditions or LPS in either the CX3CR1^KO^ (NMX 5.553 ± 0.339%, HPX 6.203 ± 0.928%, HPX + LPS 5.860 ± 1.508%), FKN^KO^ retinas (NMX 5.823 ± 0.715%, HPX 7.035 ± 0.922%, HPX + LPS 6.372 ± 0.673%), CX3CR1^T281M^ retinas (NMX 5.993 ± 2.13%, HPX 5.375 ± 1.690%, HPX + LPS 5.775 ± 1.254%), or CX3CR1^V250I^ retinas (NMX 6.440 ± 0752%, HPX 7.381 ± 0.928%, HPX + LPS 7.116 ± 1.168%) (Figure 3B). Evidence of fibrinogen accumulation in response to hypoxic conditions was observed in the WT (NMX 0.439 ± 0.411% vs. HPX 1.447 ± 1.078% *p* = 0.0131, NMX vs. NMX + LPS 1.738 ± 0.823% = 0.0013) and in FKN^KO^ retinas (NMX 0.261 ± 0.155% vs. HPX 0.792 ± 0.288% *p* = 0.0018, NMX vs. HPX + LPS 1.030 ± 0.419% *p* < 0.0001), but the addition of LPS in neither group showed exacerbated fibrinogen accumulation. The CX3CR1^KO^ retinas showed an increase in fibrinogen in response to LPS administration but not hypoxia alone (NMX 0.509 ± 0.270% vs. HPX 1.265 ± 0.687% *p* = 0.0656, NMX vs. HYP + LPS 1.966 ± 1.002% *p* = 0.0003, HPX vs. HPX + LPS *p* = 0.0931). Interestingly, neither the CX3CR1^T281M^ tissues (NMX 0.4527 ± 0.5687%, HPX 0.706 ± 0.368%, HPX + LPS 0.3852 ± 0.160%) nor CX3CR1^V250I^ (NMX 0.592 ± 0.338%, HPX 0.561 ± 0.492%, HPX + LPS 0.762 ± 0.378%) tissues showed a change in fibrinogen accumulation to any experimental conditions (Figure 3C). These data suggest that neuronal regulation of microglia influences the fibrinogen response to the combinatorial effects of hypoxia and systemic inflammation.

**Figure 4:**
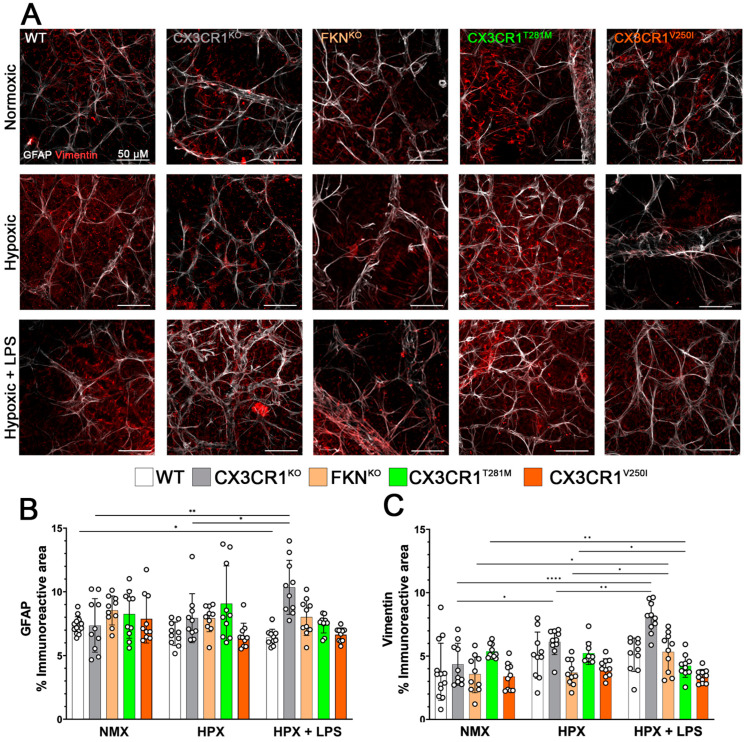
GFAP immunoreactivity decreases in response to hypoxia-induced retinopathy in WT but not CX3CR1^KO^ or FKN^KO^ retinas. (**A**) Representative 40× magnification confocal images of mouse retinas stained with astrocyte marker GFAP (white) and Müller glia marker vimentin (red). (**B**,**C**) Quantification of GFAP and vimentin immunoreactive areas, respectively. Data show mean ± SD, n = 10 mice per group, dots indicate average for individual mice. Scale bar measures 50 μm. * *p* < 0.05, ** *p* < 0.01, **** *p* < 0.0001 using one-way ANOVA with multiple comparisons and Tukey’s correction.

With evidence of reactive microgliosis and fibrinogen accumulation, in response to hypoxic conditions in the CX3CR1^KO^ and FKN^KO^ retinas, we hypothesized that there would be a corresponding phenotype of macroglia reactivity. To quantify macroglia responses, tissues were stained for astrocytes (GFAP, white) and Müller glia (vimentin, red) (Figure 4A). In the WT mice, we did not observe a change in GFAP immunoreactivity in response to hypoxic conditions (NMX 7.364 ± 0.677% vs. HPX 6.708 ± 0.916% *p* = 0.1109); however, GFAP immunoreactivity decreased in our two-hit model (NMX vs. HPX + LPS 6.4356 ± 0.6419% *p* = 0.0165). The opposite effect was observed in the CX3CR1^KO^ tissues with a significant increase in the GFAP immunoreactive area to our two-hit model (NMX 7.364 ± 2.096% vs. HPX + LPS 10.349 ± 2.123% *p* = 0.0035), but not hypoxia alone (NMX vs. HPX 7.929 ± 1.918% *p* = 0.0082). There was no significant change in GFAP expression observed in the FKN^KO^ (NMX 8.542 ± 1.127%, HPX 7.909 ± 1.032%, HPX + LPS 8.004 ± 1.423%), CX3CR1^T281M^ (NMX 8.242 ± 1.880%, HPX 9.061 ± 2.974%, HPX + LPS 7.488 ± 0.7083%), or CX3CR1^V250I^ (NMX 7.867 ± 1.881%, HPX 6.551 ± 0.999%, HPX + LPS 6.635 ± 0.543%) tissues (Figure 4B). When testing the Müller glia response to hypoxic conditions, there were no statistically significant changes in vimentin immunoreactivity in the WT (NMX 3.764 ± 2.259%, HPX 5.074 ± 1.828%, HPX + LPS 5.106 ± 1.324%) or in the CX3CR1V2501 (NMX 3.382 ± 1.074%, HPX 4.072 ± 0.723%, HPX + LPS 3.403 ± 0.542%) retinas (Figure 4B). However, there was evidence of increased vimentin immunoreactivity in CX3CR1^KO^ in response to hypoxia and exacerbated in our two-hit model (NMX 24.352 ± 1.659% vs. HPX 6.121 ± 1.007% *p* = 0.0153, NMX vs. HPX + LPS 8.021 ± 1.2000% *p* < 0.0001, HPX vs. HPX + LPS *p* = 0.0090). The FKN^KO^ retinas showed an increase in the vimentin immunoreactive area in response to LPS (NMX 3.581 ± 1.474% vs. HPX 3.689 ± 1.010%, NMX vs. HPX + LPS 5.333 ± 1.551% *p* = 0.0210, HPX vs. HPX + LPS *p* = 0.0324) but not hypoxia. In the receptor-defective CX3CR1^T281M^ tissues, we observed the opposite effect with a decrease in the vimentin immunoreactive area in response to LPS but not to hypoxia alone (NMX 5.368 ± 0.5585% vs. HPX 5.223, NMX vs. HPX + LPS 4.229 ± 0.9039% *p* = 0.0097, HPX vs. HPX + LPS 0.0254). However, the CX3CR1^V250I^ retinas showed no change in vimentin immunoreactivity (NMX 3.382 ± 1.074%, HPX 4.072 ± 0.7230, HPX + LPS 3.404 ± 05428% (Figure 4C). Together, these data suggest that hypoxia induces microglial reactivity and vascular pathology in all our models of microglial regulation. However, in models of microglia dysregulation, there was an increase in the Müller glia and astrocyte response. Interestingly, while we observed an increase in the endothelial cell marker CD31 in the WT mice, it was not observed in any other mouse models.

### 2.3. CX3CR1 Regulation Affects the Apoptosis, Adaptive Immune, and Extracellular Matrix Organization Responses to Hypoxia-Induced Retinopathy

**Figure 5:**
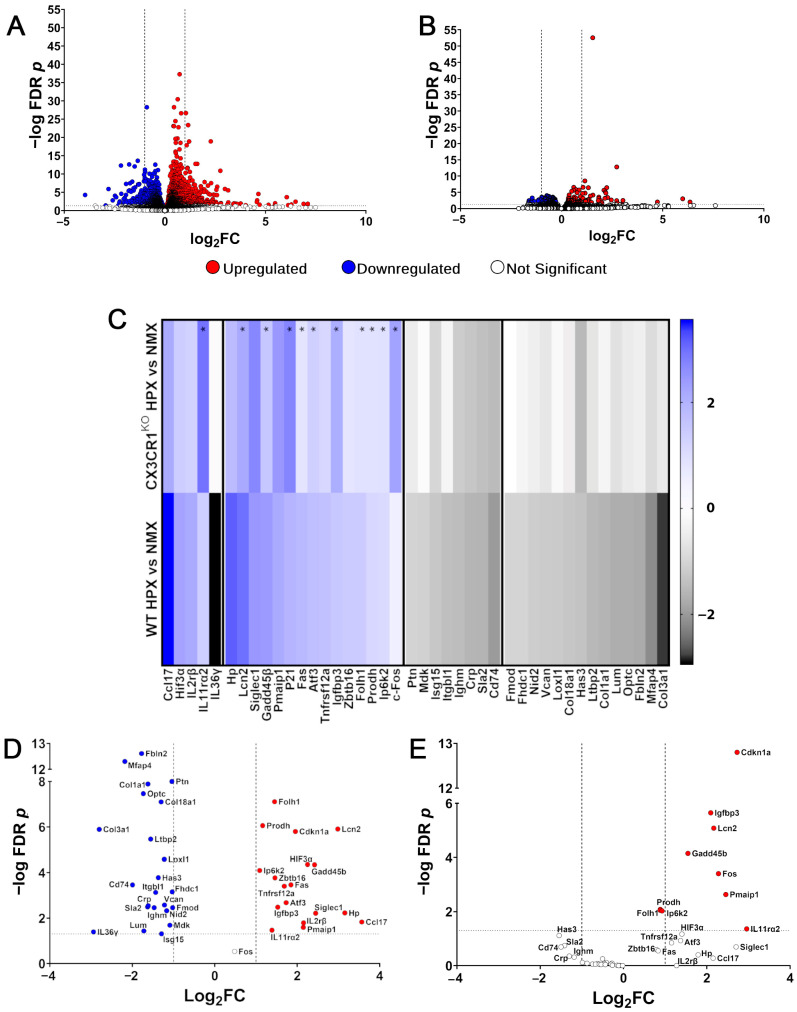
DEG analysis of bulk retinal RNAseq shows immunomodulation and decreased ECM transcripts in response to hypoxia in WT but not CX3CR1^KO^ retinas. (**A**,**B**) Volcano plot of all differentially expressed genes in WT (**A**) and CX3CR1^KO^ (**B**) hypoxic vs. normoxic samples. Dashed horizontal line represents the FDR p-value cutoff of 0.05 while dashed vertical threshold lines indicate log2FC > 1 and <−1. (**C**) Heat map showing inflammatory/hypoxic, apoptosis, adaptive immune, and ECM responses. Asterisks (*) indicate genes where FDR *p* < 0.05 in the CX3CR1^KO^ samples. C-FOS in the WT cohort did not show a significant change in expression. (**D**,**E**) Volcano plot showing genes associated with inflammatory, hypoxic, apoptosis, adaptive immune, and ECM responses in WT (**D**) and CX3CR1^KO^ (**E**) groups. Red points represent upregulated genes and blue points represent downregulated genes when FDR *p* < 0.05 while white points represent genes with FDR *p* > 0.05.

Our data suggest that the macroglial responses to hypoxia-induced retinopathy are significantly affected by dysregulation of the FKN-CX3CR1 signaling axis. To understand the mechanisms driving this phenotype, we conducted bulk RNA sequencing on the control and hypoxic WT and CX3CR1^KO^ retinas. These groups were selected because of the observed decrease in GFAP immunoreactivity in the WT retinas but increased GFAP astrogliosis in the CX3CR1^KO^ retinas in our model of hypoxia-induced retinopathy. We were also interested in characterizing the effects of hypoxia without additional inflammatory signals generated by endotoxemia. Here, we found a greater number of genes differentially expressed in the WT retinas in response to hypoxia (975 genes upregulated and 1516 genes downregulated vs. CTR (Figure 5A) compared to the CX3CR1^KO^ retinas (105 genes upregulated and 114 genes downregulated vs. CTR (Figure 5B). This may indicate that CX3CR1 deletion has a stronger overall effect on the retinal transcriptome than short-duration hypoxia.

We found evidence of an immunomodulatory response in the WT mice with CCL17 upregulation (WT log_2_ fold change (log_2_FC) = 3.56, FDR *p* = 0.0148) along with cytokine receptor IL2rβ (log_2_FC = 2.13, FDR *p* = 0.0161) and a decrease in IL-1 superfamily member IL36γ expression (IL36γ, log_2_FC = −2.94, FDR *p* = 0.0404) (Figure 5C–E and Supplementary Table S1). We did not observe a similar response in the CX3CR1^KO^ tissues (log_2_FC = 2.15, FDR *p* = 0.5310, IL2rβ log_2_FC = 1.28, FDR *p* = N/A, IL36γ log_2_FC = −0.12, FDR *p* = 0.9946). In both the WT and CX3CR1^KO^ tissues, the receptor for hypoxia-inducible IL-11 [21] was upregulated in response to hypoxia (WT log_2_FC = 1.39, FDR *p* = 0.0340, CX3CR1^KO^ log_2_FC = 2.96, FDR *p* = 0.0436). Next, we looked at how microglia dysregulation affects the expression of the hypoxia-inducible factor (HIF) family of transcription factors. The WT tissues showed an increase in expression of HIF-2α, which was statistically significantly upregulated (log_2_FC = 0.48, FDR *p* = 0.0001), and HIF-3α (log_2_FC = 2.25, FDR *p* < 0.0001), but not HIF-1α (log_2_FC = 0.1, FDR *p* = 0.6101). The CX3CR1^KO^ tissues did not show a significant change in the expression of any HIF transcripts (HIF-3α log_2_FC= 1.40, FDR *p* = 0.0675, HIF-1α log_2_FC = 0.05, FDR *p* = 0.9701, HIF-2α log_2_FC = 0.26, FDR *p* = 0.6093). These results suggest that exacerbated microglia reactivity to hypoxic conditions significantly alters the regulation of HIF proteins.

**Figure 6:**
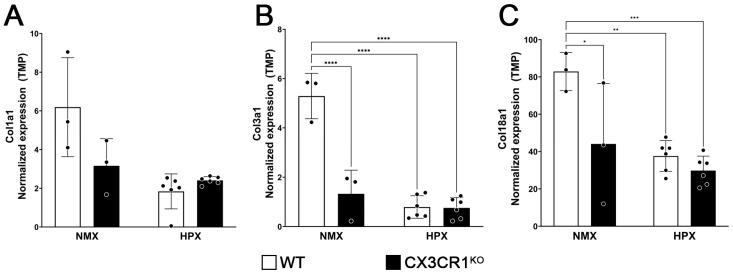
Decreased transcript abundance of type 1, 3, and 18 collagens observed in WT hypoxic and CX3CR1^KO^ retinas compared to WT control tissues. (**A**–**C**) Comparison of unique transcript counts mapped to collagen genes for normoxic and hypoxic WT (white) and CX3CR1^KO^ (black). (**A**) Collagen 1a1. (**B**) Collagen 3a1. (**C**) Collagen 18a1. Data show mean ± SD, n = 3 control, and n = 6 hypoxic mice per group, dots indicate average for individual mice. * *p* < 0.05, ** *p* < 0.01, *** *p* < 0.001, **** *p* < 0.0001 using two-way ANOVA with multiple comparisons and Tukey’s correction.

We next sought to identify gene pathways involved in the hypoxic response by performing pathway analysis utilizing genes that met our statistical (FDR *p* < 0.05) and biological (log_2_ > 1, <−1) cutoffs. In the CX3CR1^KO^ hypoxic samples, we found that both the metabolic and cell cycle pathways were upregulated; however, due to the low number of genes that met our cutoff criteria, no downregulated pathways were identified. Instead, we focused our analysis on the hypoxic responses in the WT tissues, specifically on pathways associated with neuroinflammatory responses, apoptosis, immune responses, and angiogenesis (Figure 5C–E, Supplementary Table S1). While many genes associated with apoptosis were upregulated in both the WT and CX3CR1^KO^ hypoxic samples, microglia dysregulation does appear to affect the apoptotic response with an increase in FAS transcripts in the WT but not CX3CR1^KO^ retinas (WT log_2_FC= 1.85, FDR *p* = 0.0003, CX3CR1^KO^ log_2_FC = 0.83, FDR *p* = 0.28) while c-FOS expression was increased in the CX3CR1^KO^ but not WT retinas (CX3CR1^KO^ log_2_FC = 2.28, FDR *p* = 0.000392, WT log_2_FC = 0.48, FDR *p* = 0.29). The WT hypoxic retinas also showed a unique profile of downregulation of genes associated with the adaptive immune responses not observed in the CX3CR1^KO^ tissues (Figure 5C–E, Supplementary Table S1). Also unique to the hypoxic response in the WT retinas was a decrease in expression in ECM organization proteins, a process required to make tissues permissive for angiogenesis (Figure 5C, Supplementary Table S1) [22]. Included in this pathway are type 1, 3, and 18 collagen components of elastic fibers, which maintain the lateral strength of the vasculature. However, it was surprising that there was not a significant decrease in collagen expression in the hypoxic CX3CR1^KO^ retinas, given the evidence of fibrinogen accumulation in those tissues. Examining the normalized abundance of collagen transcripts (transcripts per million, TPM), we found that under normoxic conditions, the CX3CR1^KO^ retinas expressed significantly decreased levels of type I collagen (WT 6.129 ± 2.560 TPM, CX3CR1^KO^ 3.16 ± 1.402 TPM, two-way ANOVA *p* = 0.0413), type III collagen (WT 5.292 ± 0.921 TPM, CX3CR1^KO^ 1.326 ± 0.958 TPM, two-way ANOVA *p* < 0.0001), and type XVIII collagen (WT 82.905 ± 10.216 TPM, CX3CR1^KO^ 44.087 ± 32.388 TPM, two-way ANOVA *p* = 0.0252) (Figure 6A–C). Also, under hypoxic conditions, there was no significant change in the normalized transcript abundance of these collagen genes in the CX3CR1^KO^ retinas. These data suggest disruption in the FKN-CX3CR1 signaling axis alters the immune and apoptotic response to hypoxia-induced retinopathy.

### 2.4. Hypoxia Induces a Reduction in Endostatin Immunoreactivity

**Figure 7:**
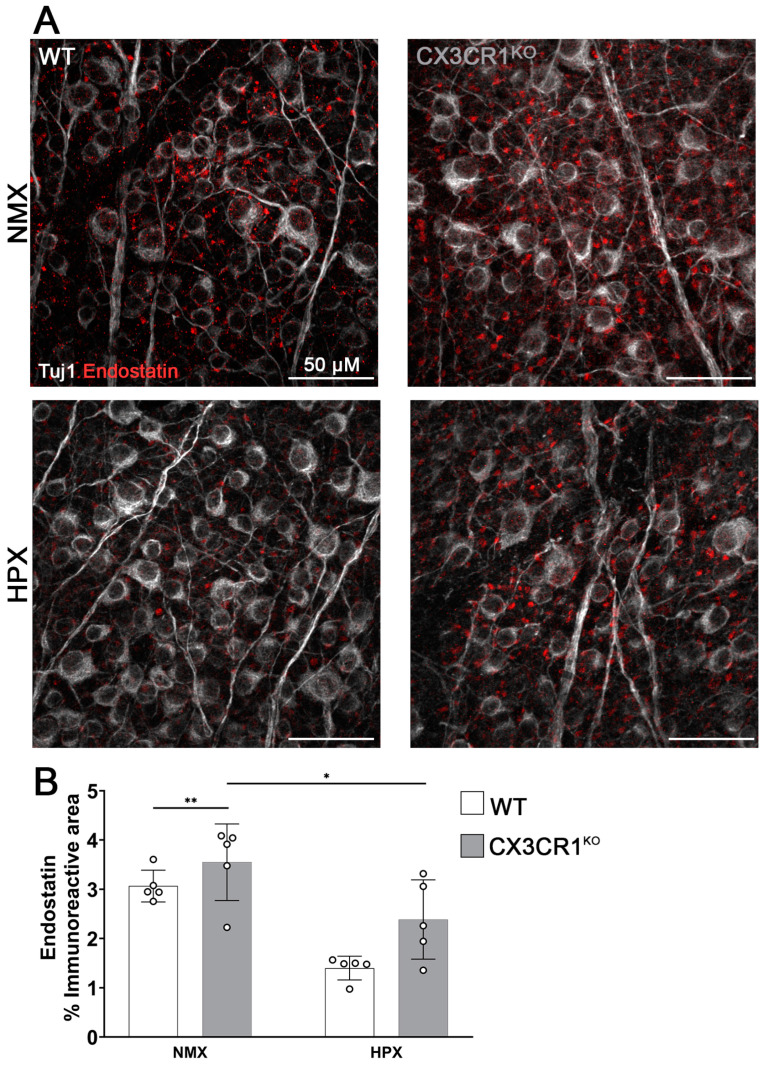
Hypoxia induces decreased expression of endostatin in both WT and CX3CR1^KO^ retinas. (**A**) Representative 40× magnification confocal images of mouse retinas stained with RGC axonal marker Tuj1 (white) and endostatin (red). (**B**) Quantification of endostatin immunoreactive area. Data show mean ± SD, n = 5 mice per group, dots indicate average for individual mice. Scale bar measures 50 μm. * *p* < 0.05, ** *p* < 0.01 using two-way ANOVA with multiple comparisons and Tukey’s correction.

Endostatin is a 20 kD cleavage product of type XVIII collagen and functions as a potent inhibitor of angiogenesis, preventing vascular budding by sterically blocking vascular endothelial growth factor receptor binding to its ligand [23,24]. Since we observed decreased transcript abundance of type XVIII collagen in the CX3CR1^KO^ mice compared to the WT retinas under normoxic conditions and a reduction in the hypoxic WT retinas compared to their normoxic control, we hypothesized that the accumulation of endostatin in the retina would also be reduced. To quantify changes in endostatin expression, retinal leaflets from the WT and CX3CR1^KO^ mice were stained with anti-endostatin antibodies (red) (Figure 7A,B). In the mouse retina, endostatin accumulates in bipolar cells, where it localizes to both cell bodies and axonal end processes of bipolar cells [25]. Confocal imaging identifies this as puncta in the nerve fiber layer (NFL) and diffuses deeper where bipolar axons terminate in flat-mounted tissues. Thus, to quantify changes in endostatin expression, we focused on endostatin accumulation in bipolar cell bodies in the NFL using Tuj1 immunostaining (white), an RGC axonal marker, as an anatomical marker for the NFL.

Firstly, we tested if there was a difference in endostatin accumulation between the WT and CX3CR1^KO^ retinas. Here, we found that there was no statistical difference in the endostatin immunoreactive area (WT control 3.07 ± 0.323%, CX3CR1^KO^ control 3.55 ± 0.778%, two-way ANOVA *p* = 0.9759).

However, we found that hypoxia induced a significant reduction in endostatin accumulation in the WT (1.4 ± 0.241%, WT control vs. WT hypoxic two-way ANOVA *p* = 0.0063) and CX3CR1^KO^ tissues (2.387 ± 0.805%, CX3CR1^KO^ control vs. CX3CR1^KO^ hypoxic two-way ANOVA *p* = 0.032). We also tested how systemic inflammation affected retinal endostatin responses (Supplementary Figure S1A,B). We found that LPS alone did not affect endostatin expression (WT LPS 2.095 ± 0.396, vs. WT control two-way ANOVA *p* = 0.0912, CX3CR1^KO^ LPS 3.192 ± 0.178, vs. CX3CR1^KO^ control two-way ANOVA *p* = 0.9997). However, the addition of LPS to hypoxia increased endostatin accumulation in the WT tissues (WT hypoxia with LPS 2.811 ± 0.258, vs. WT hypoxia two-way ANOVA *p* = 0.0018) while in the CX3CR1^KO^ tissues, endostatin expression was similar to hypoxia alone (CX3CR1^KO^ hypoxia with LPS 2.414 ± 0.461 vs. CX3CR1^KO^ hypoxia two-way ANOVA *p* = 0.9999 vs. CX3CR1^KO^ control two-way ANOVA *p* = 0.0223). Lastly, we tested to see if endostatin expression is altered in diabetic patients (Supplementary Figure S1C,D). Here, we found that endostatin expression in human tissues was unchanged in diabetic patients (non-diabetic 4.681 ± 1.778%, diabetic 3.845 ± 1.463%, Student’s T-test *p* = 0.4595). These data suggest that while hypoxia reduces endostatin accumulation in retinal tissue, inflammation exerts an opposing effect in the mouse retina.

### 2.5. Hypoxia Ameliorates the LPS-Induced Inflammatory Cytokine Response

**Figure 8:**
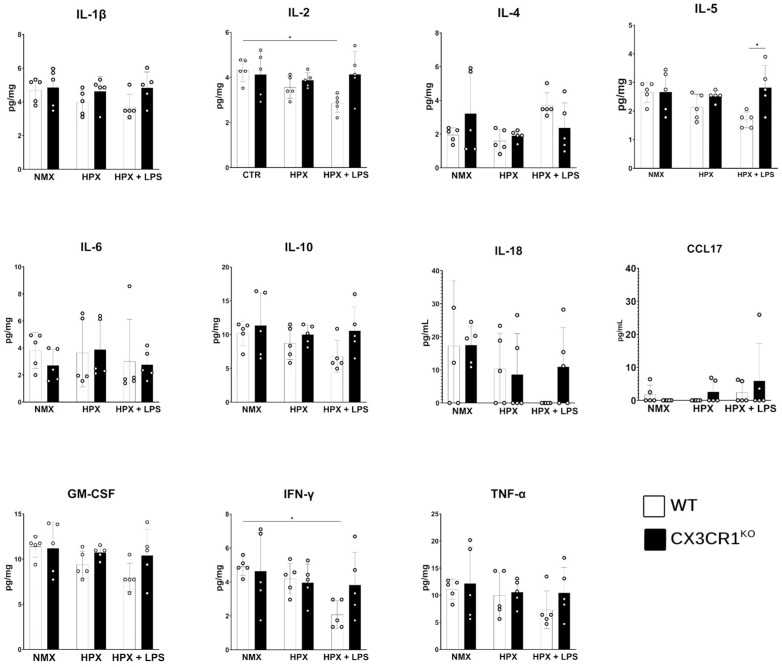
Hypoxia inhibits inflammatory IL-2 and IFN-γ cytokine responses to LPS in WT (white) but not CX3CR1^KO^ (black) CNS lysates. * *p* < 0.05 using two-way ANOVA with multiple comparisons and Tukey’s correction.

We next sought to test for changes in cytokine responses in CNS tissue. Brain lysate samples were utilized as both hypoxia and LPS treatments act systemically on CNS tissues [26,27,28,29]. We tested for changes in the expression of pro-inflammatory cytokines IL-1β, IL-2, IL-6, IFN-γ, and TNF-α; IL-18, regulator of hypoxia; GM-CSF, a marker of microglia proliferation; and anti-inflammatory cytokines IL-4, IL-5, IL-10, and CCL17 in WT and CX3CR1^KO^. When comparing cytokine expression levels between the control and hypoxic groups, we found that there was no significant change in expression in any of the analytes (Figure 8). We observed in the WT mice under conditions of hypoxia and LPS a significant reduction in IL-2 (control 4.32 ± 0.72 pg/mg, hypoxia with LPS 2.85 ± 0.42 pg/mg, two-way ANOVA *p* = 0.0269) and IFN-γ (control 4.91 ± 0.52 pg/mg, hypoxia with LPS 2.07 ± 0.83 pg/mg, two-way ANOVA *p* = 0.0419). The CX3CR1^KO^ samples showed no change in cytokine expression in response to the two-hit model of hypoxia with LPS.

## 3. Discussion

We found that while hypoxia induces significant microglia reactivity in WT tissues, the microglia responses were altered in models of microglia dysregulation. When the CX3CR1-FKN signaling axis is blocked in CX3CR1^KO^ and FKN^KO^ tissues, we observed that our two-hit model induced an exacerbation of microglial reactivity. However, in signaling defective models, LPS failed to induce a similar exacerbation of microglia reactivity. This finding may shed light on the conflicting results reported in studies examining the association between CX3CR1 polymorphisms and susceptibility and severity in human disease [30,31,32,33,34].

We also found that the anti-inflammatory response to hypoxia is affected by microglia dysregulation, with the WT but not CX3CR1^KO^ retinas showing an increase in anti-inflammatory CCL17 and a decrease in IL-1 superfamily member IL-36γ retinal transcripts; however, we did not see a change in CCL17 protein expression in the brain [35,36]. This may explain why we observed an increase in macroglia reactivity in response to the combination of systemic inflammation and hypoxic conditions in the CX3CR1^KO^ but not WT tissues. Despite evidence of microglia reactivity in all of the animal models utilized in this study, we did not observe evidence of neuronal loss in response to hypoxia or our two-hit model. We hypothesize that this is due to the short 7-day duration of hypoxic conditions being insufficient to induce enough stress in neuronal cells [37].

When considering the vascular response to hypoxia, we observed a significant increase in CD31 immunoreactivity in the WT tissues but not in any retinas from animal models of defective microglia regulation. Based on our transcriptional data, we hypothesize that exacerbated microglial reactivity in the CX3CR1^KO^ retinas dysregulates the ECM response to hypoxia, reducing the permissive environment for angiogenesis. Interestingly, this mechanism may not involve endostatin as we observed a similar reduction in endostatin accumulation in both the WT and CX3CR1^KO^ retinas in response to hypoxia in tissues despite transcript abundance of endostatin precursor type XVIII collagen in normoxic CX3CR1^KO^ tissues being similar to hypoxic WT tissues. Endostatin accumulation in retinal tissues does appear to be strongly affected by inflammatory responses. However, the mechanisms driving this response have yet to be elucidated.

We also observed that microglia dysregulation influenced the expression of apoptosis-associated genes. The WT hypoxic retinas showed an increase in FAS in the WT tissues but not in CX3CR1^KO^, while FOS, an apoptosis-associated gene that also has reported neuroprotective effects [38], was upregulated in the CX3CR1^KO^ but not in WT retinas. This finding is surprising, as hypoxia is typically associated with neuroprotective responses. However, it could be explained by the decrease in IL-36γ as IL-1 has been reported to induce c-fos expression [39].

LPS induces inflammatory responses that are well characterized in the CNS where it induces an inflammatory response with increased expression of cytokines such as IL-1β, IL-6, IFN-γ, and TNF-α [27,40,41]. The effects of endotoxemia on the hypoxic mouse brain have been reported to induce exacerbation of inflammatory mediators IL-1β, IL-6, and TNF-α [39]. Here, we report that the combination of systemic endotoxemia in a more severe (7.5% O_2_ vs. 10.16% O_2_) and longer duration (7 days vs. 24 h) significantly alters the inflammatory response to LPS-mediated hypoxia. We observed no change in expression in IL-1β, IL-6, and TNF-α in either the WT or CX3CR1^KO^ retinas and instead observed a significant decrease in IL-2 and IFN-γ expression in the WT hypoxic mice. The shift in response could be explained that by day 7 of hypoxia, there is a transition from an acute HIF-1α/2α response to a chronic response characterized by HIF-3α upregulation [42]. While the exact functions of HIF-3α are not as elucidated as other HIF family members, it is reported to be a negative regulator of HIF-1α/2α, a promotor of apoptosis and regulator of angiogenesis [43,44]. Interestingly, microglia dysregulation significantly alters the expression of the HIF family of transcription factors under hypoxic conditions, suggesting that inflammatory responses may co-regulate the expression of the HIF family of transcription factors. We also found no evidence of altered expression of cytokines associated with HIF-1α or HIF-2α responses when we examined the expression of IL -18 or IL-1β, cytokines involved in the HIF-1α response, and IL-4, a downstream response to HIF-2α, in either WT or CX3CR1^KO^ in CNS lysates [45,46]. Instead, we found that the WT hypoxic retinas showed a decrease in LPS-responsive inflammatory mediators IL-2 and IFN-γ. This suggests that chronic hypoxia and HIF-3α exert an inflammatory dampening response to endotoxemia that is dependent on microglia responses.

It is well documented that high levels of circulating LPS are found in diabetic patients. Hence, those studies point to a strong correlation between systemic LPS challenge and the progression of DR [6,47,48]. Our results suggest that if inflammatory input from diabetes was ameliorated, be it through resolving the underlying diabetic condition, via anti-inflammatory or anti-angiogenic treatments, the remaining hypoxic pathology would not exacerbate retinal pathology. HIF1α is commonly reported as a marker of DR both for diagnosis and prognosis while HIF2a is involved in neovascularization [49,50]. As both HIF1α and 2α are negative regulators of HIF3α, we hypothesize that anti-inflammatory treatment in conjunction with drugs such as phloretin [51] that induce HIF3α responses may show a significant improvement in DR pathology than anti-inflammatory treatment alone.

## 4. Materials and Methods

### 4.1. Animals

All mice utilized in this study were of the C57BL/6J background. Wild-type (WT) (Jackson Laboratories, Bar Harbor, ME, USA, Strain # 000664) and CX3CR1^GFP/GFP^ (CX3CR1^KO^) were sourced from Jackson Laboratories (Bar Harbor, ME, USA, Strain # 005582). Mice expressing human CX3CR1 polymorphisms I249 and T280 in mouse CX3CR1 gene, mCX3CR1^V250I,^ and mCX3CR1^T281M^ were generated by Biogen© (Cambridge, MA, USA). CX3CR1 polymorphic variant mice utilized in this experiment were heterozygous for the mutant allele, expressing either mCX3CR1^V250I/WT^ (CX3CR1^V250I^) and mCX3CR1^T281M/WT^ (CX3CR1^T281M^). CX3CL1^–/–^ (FKN^KO^) were obtained from Dr. Sergio Lira (Icahn School of Medicine at Mount Sinai) and mice were bred at the University of Texas at San Antonio facilities. Both male and female mice were utilized in this study. Animal protocols were consistent with Association for Assessment and Accreditation of Laboratory Animal Care (AAALAC) standards, approved by the UTSA-Institutional Animal Care and Use Committee, and in accordance with NIH guidelines.

### 4.2. Two-Hit Model of Hypoxia-Induced Retinopathy and Systemic Inflammation

Adult (6–10 week) male and female mice were exposed to hypoxic conditions of 7.5% oxygen for 7 days in an airtight chamber (Coy Laboratory Products, Grass Lake, MI, USA, InVivo Cabinet model 15) in open-air housing cages with free access to food and water. Oxygen levels were maintained by injection of supplemental N_2_ and O_2_ gas. Animals were monitored twice daily and returned to normoxic conditions on an as-needed basis for periods no longer than 30 min for injections or welfare concerns. To recapitulate the systemic inflammation and endotoxemia reported in human diabetic patients, cohorts of hypoxic mice were administered 0.08 mg/Kg of LPS (Sigma Aldrich, St. Louis, MO, USA, #L2637) or PBS vehicle control on days 6 and 7 of the experiment [2,5,6,52] (Supplemental Figure S1). Age-matched control animals were maintained under normoxic conditions.

### 4.3. Streptozotocin-Induced Hyperglycemia

Hyperglycemia was induced in WT male mice (n = 5) by I.P. injection of 60 mg/Kg STZ (Sigma Aldrich, St. Louis, MO, USA, #S0130) in citrate buffer daily for 5 days [2,5,6,52]. Female mice do not develop consistent levels of hyperglycemia in response to this dose of streptozotocin (STZ) due to the antidiabetic actions elicited by 17β-estradiol as described previously [52]. Blood glucose was monitored weekly for 10 weeks. Animals were considered hyperglycemic with sustained blood glucose levels > 250 mg/dL.

### 4.4. Tissue Collection

Anesthetized mice were transcardially perfused with cold 1× Hanks’s Balanced Salt Solution (HBSS, Fisher Scientific, Pittsburgh, PA, USA, #BW10-547F). Enucleated whole globes were fixed in 4% paraformaldehyde (PFA, Sigma Aldrich, St. Louis, MO, USA, #P6148) for 20 min. Retinas were then dissected and transferred along with optic nerves to 1% PFA. Brains collected for histology were fixed overnight in 4% PFA. Fixed tissues were then prepared for long-term storage as previously described [52]. Briefly, fixed tissues prepared for storage first by incubating them overnight in a cryoprotection solution (200 mL glycerol, 200 mL 0.4 M Sorenson’s buffer, and 600 mL water) at 4 °C then transferred to a cryoprotection solution (500 mL 0.2 M PO_4_, 10 g PVP-40, 300 g sucrose, and 300 mL ethylene glycol) overnight at 4 °C, then were stored at −20 °C. Protein collection from brains was performed by mechanical homogenization in a protein extraction buffer (9.15 mL water, 0.6 mL 2.5 M NaCl, 0.1 mL 1 M Tris base, 20 μL 0.5M EDTA, and 100 μL of protease inhibitor cocktail (Millipore Sigma, Burlington, MA, USA, #0469311600) and protein supernatant was collected by centrifugation at 4 °C, 12,000 RPM for 15 min then stored at −80 °C. Plasma was collected from peripheral blood obtained by submandibular vein puncture in EDTA-treated tubes (BD, Franklin Lakes, NJ, USA, #365974). Whole blood was centrifuged for 20 min at 2000g at 4 °C. Plasma was transferred to sterile 0.6mL tubes with 1 μL of protease inhibitor cocktail per 100 μL of plasma then stored at −80 °C.

### 4.5. Retinal RNA Collection and Processing

Retinas were homogenized in 800 μL ice-cold Trizol reagent (Thermo Fisher Scientific, Pittsburgh, PA, USA, #15596026) following the manufacturer’s protocol modified with an overnight precipitation at −20 °C then resuspended in 20 μL of RNase-/DNase-free water (Thermo Fisher Scientific, Pittsburgh, PA, USA, 10977-015). Sample cleanup and residual DNA was removed utilizing RNeasy (Qiagen, Germantown, MD, USA, #74104) kits with on-column DNase digestion per manufacturer’s protocol. QA/QC of samples, library prep, and sequencing services were performed at the UT Health Genome Sequencing Facility (UT Health San Antonio, Greehey Children’s Cancer Research Institute, San Antonio, TX, USA). Sequencing data were processed at The UTSA Genomics Core (University of Texas at San Antonio, San Antonio, TX, USA) utilizing the CLC Genomics Workbench (Qiagen, Germantown, MD, USA, #832021) platform. Differential gene expression (DGE) was calculated utilizing the DESeq2 [53] package from Bioconductor [54] in Rstudio (RStudio version 4.3.3, Boston, MA, USA, https://www.rstudio.com). Transcripts were filtered for significance with a false detection rate (FDR)-adjusted *p* cutoff of <0.05; non-significant results (FDR *p* > 0.05) were omitted from downstream analyses. Gene ontology was performed using Metascape [55] using genes that were highly up- or downregulated (log2FC > 1 or <−1) while genes failing to meet this threshold were omitted. Cohorts of control (N = 3) WT and CX3CR1^KO^ animals and hypoxic (N = 6) WT and CX3CR1^KO^ animals were utilized in this experiment.

### 4.6. Immunofluorescent Staining

Tissues were prepared utilizing protocols described previously [37,51]. In brief, retinas were cut into 4 radial pieces and selected at random to be stained for markers of interest. Tissues were blocked overnight in blocking solution (450 μL 10% normal goat serum, 450 μL 10% normal donkey serum, and 100 μL of 10% triton/mL) at 4 °C. Then, tissues were incubated overnight with primary antibodies (Table 1) diluted in fresh blocking solution. Unbound primary antibodies were removed by 5 washes of 5 min each in PBT with 0.1% Triton at room temperature. Visualization of primary antibodies was performed with host-specific secondaries incubated for 2 h at room temperature (Table 1); indirect staining of vimentin utilized a biotinylated anti-chicken secondary subsequently visualized with streptavidin-conjugated Cy3. Tissues were again washed as described previously then nuclei were stained with Hoechst 3342 (Thermo Fisher Scientific #H1399) for 7 min at room temperature. Unbound Hoechst was removed from tissues by washing 5 times in PBS before tissues were mounted on superfrost plus microscope slides (Fisher Scientific, Pittsburgh, PA, USA, #12-550-15) and mounted in Fluorsave reagent (Millipore Sigma, Burlington, MA, USA, #345789).

### 4.7. Tissue Imaging and Quantification

Six images were obtained per tissue at 40× on a Zeiss 710 NLO 2P confocal microscope at the UTSA Cell Analysis Core (University of Texas at San Antonio, San Antonio, TX, USA) with three images taken from both the central (inner 1/3rd of the retinal leaflet) and peripheral (outer 1/3rd of the retinal leaflet) regions of the tissue while avoiding edge effects and sites of tissue damage from processing. Image analysis and processing were performed utilizing IMARIS (Oxford Instruments, Abingdon, Oxfordshire, UK, version 6.4), Photoshop (Adobe, San Jose, CA, USA, version 22.1), and ImageJ software (Fiji version 1.53f53). Cell densities were quantified using the object counter tool in Photoshop and ImageJ. Immunoreactive area was calculated by converting images in ImageJ to 32-bit grayscale images and manual thresholds set to match raw image intensity. Microglia reactivity was calculated using the transformation index (TI) as described [56]. In brief, microglia were traced using ImageJ to determine perimeter and area, then TI was calculated with the following equation: perimeter^2^/(4π × area^2^). To best report the effect of treatment on microglia morphology, TI values were plotted as individual values (N > 6 per animal) not averaged to each animal [57].

### 4.8. Quantification of Endostatin in Human Retinal Tissue

Post-mortem human retinas were obtained from the National Disease Research Interchange (NDRI, Philadelphia, PA, USA) and prepared as described previously [58]. A total of 10 tissues were obtained and grouped as diabetic (6) or non-diabetic (4) based on provided patient diagnostic information. Two retinal leaflets, one each from the central and peripheral regions, from each tissue were stained. Staining was as described previously except the primary antibody incubation time was increased to 72 h and the secondary antibody incubation times were increased to 24 h.

### 4.9. Statistical Analysis

Statistical significance for differentially expressed genes was generated by DESeq2 [53]. All other statistical analyses utilized GraphPad Prism v9.5.1. Statistical significance indicators are denoted as the following: * *p* < 0.05, ** *p* < 0.01, *** *p* < 0.001, and **** *p* < 0.0001. Comparisons between two data elements utilized a two-tailed parametric Student’s *t*-test with Welch’s correction. One-way ANOVAs were utilized when comparing a group with 3 or more elements, while two-way ANOVAs were utilized when comparing multiple groups containing multiple elements. All ANOVAs utilized Tukey’s post-hoc test and multiple comparisons.

## 5. Conclusions

These findings support our other studies that dysregulation of the FKN-CX3CR1 signaling axis exacerbates the inflammatory response in models of CNS disease. Further, it suggests that if we can limit the inflammatory response in a disease with a pathology involving hypoxia-overexpressed transcripts, there is the potential to promote anti-inflammatory cellular responses to further limit retinal pathology. This study also raises several questions for further investigation. Firstly, it is unclear if hypoxia-induced retinal damage correlates with alterations in visual acuity in models of microglia dysregulation. Understanding the relationship between endotoxemia and endostatin expression driving the anti-inflammatory response to endotoxemia in the hypoxic retina may provide novel insights to mitigate vascular damage.

## Figures and Tables

**Table 1:** List of antibodies utilized in this project.

Primary Antibodies				
Target	Host ^1^	RRID	Dilution	Cellular target
Anti-ionized calcium-binding adaptor molecule-1 (Iba1)	Rb	AB_839504	1:4000	Microglia
Anti-neuronal nuclei (NeuN)	Ms	AB_2298772	1:4000	Retinal ganglion cells
Anti-glial fibrillary acidic protein (GFAP)	Rt	AB_2532994	1:4000	Astrocytes
Anti-platelet endothelial cell adhesion molecule (PECAM-1/CD31)	Rt	AB_393571	1:500	Endothelial cells
Anti-fibrinogen	Rb	AB_578481	1:2000	N/A
Anti-vimentin	Ck	AB_2216267	1:1000	Müller glia
Anti-beta tubulin III (TUJ1)	Ms	AB_10063408	1:1000	Retinal ganglion cell axons
Anti-endostatin	Rb	AB_2245014	1:200	Endostatin
Secondary Antibodies				
Target	Host	RRID	Dilution	
Anti-rabbit 488	Dk	AB_2313584	1:1000	
Anti-rabbit Cy3	Gt	AB_2338006	1:1000	
Anti-rat Cy5	Dk	AB_2340694	1:1000	
Anti-rat Cy3	Gt	AB_2338394	1:1000	
Anti-mouse Cy3	Gt	AB_2338709	1:1000	
Anti-mouse Cy3	Gt	AB_2338713	1:1000	
Anti-chicken Biotin	Dk	AB_2340363	1:1000	
SA Cy3	N/A	AB_2337244	1:1000	

^1^ Rabbit (Rb), mouse (Ms), rat, (Rt), chicken (Ck), donkey (Dk), goat (Gt).

## Data Availability

RNA sequencing data that support the findings of the study have been deposited in the Gene Expression Omnibus with accession code GSE276207. These data are currently private and will be updated to be made available upon publication. Data are available upon request for reviewing purposes. Other data that support this study are available upon request.

## Supplementary Materials

The following supporting information can be downloaded at: https://www.mdpi.com/article/10.3390/ijms26031131/s1.
