# Supplementary material for: CX3CR1–Fractalkine Dysregulation Affects Retinal GFAP Expression, Inflammatory Gene Induction, and LPS Response in a Mouse Model of Hypoxic Retinopathy
Source: Int J Mol Sci. 2025 Jan 28;26(3):1131. doi: 10.3390/ijms26031131 (PMC11817233; doi:10.3390/ijms26031131)
Supplement: Supplementary file 1 [file ijms-26-01131-s001.zip › ijms-3412841-supplementary.pdf]

Supplementary Table S1. Values* for log_2 and *p* value for DEG heatmap.

Gene ID	WT HPX vs NMX		$CX3CR1^{KO}$ HPX vs NMX	
	ΔExpression (Log_2)	FDR *p* value	ΔExpression (Log_2)	FDR *p* value
Interleukins & Hypoxic response				
Ccl17	**3.56**	**1.48E-02**	2.15	5.31E-01
Hif3α	**2.25**	**4.38E-05**	1.40	6.75E-02
IL2rβ	**2.15**	**1.61E-02**	1.27	1.00E+00
IL11rα2	**1.39**	**3.40E-02**	**2.96**	**4.36E-02**
Hif1α	0.10	6.10E-01	0.05	9.71E-01
Hif2α	**0.48**	**1.31E-04**	0.26	6.04E-01
IL36γ	**-2.94**	**4.04E-02**	-0.11	9.95E-01
Adaptive response				
Ptn	**-1.04**	**1.00E-08**	-0.50	5.61E-01
Mdk	**-1.09**	**2.04E-02**	-0.09	9.86E-01
Isg15	**-1.29**	**4.87E-02**	-0.99	7.66E-01
Itgbl1	**-1.44**	**7.38E-04**	-0.27	9.69E-01
Crp	**-1.47**	**2.85E-03**	-1.18	4.84E-01
Sla2	**-1.61**	**3.22E-03**	-1.30	4.46E-01
Cd74	**-1.63**	**3.46E-04**	-1.41	1.82E-01
Col3a1	**-2.00**	**1.28E-06**	-1.50	2.03E-01
Apoptosis				
Hp	**3.15**	**5.92E-03**	1.79	3.98E-01
Lcn2	**2.99**	**1.24E-06**	**2.17**	**8.26E-06**
Siglec1	**2.45**	**6.11E-03**	2.70	2.02E-01
Gadd45β	**2.42**	**4.48E-05**	**1.55**	**7.12E-05**
Pmaip1	**2.15**	**2.54E-02**	**2.46**	**2.31E-03**
P21	**1.96**	**1.59E-06**	**2.73**	**1.58E-13**
Fas	**1.85**	**3.44E-04**	0.83	2.81E-01
Atf3	**1.73**	**2.09E-03**	1.37	1.18E-01
Tnfrsf12a	**1.69**	**3.95E-04**	1.15	1.43E-01
Igfbp3	**1.53**	**3.31E-03**	**2.09**	**2.24E-06**
Zbtb16	**1.46**	**1.69E-04**	0.79	2.55E-01
Folh1	**1.45**	**7.76E-08**	**0.88**	**8.86E-03**
Prodh	**1.16**	**8.77E-07**	**0.88**	**8.21E-03**
Ip6k2	**1.09**	**8.01E-05**	**0.93**	**9.37E-03**
c-Fos	0.48	2.95E-01	**2.28**	**3.92E-04**

Supplementary Table S1. Continued.

Gene ID	WT HPX vs NMX		CX3CR1^{KO} HPX vs NMX	
	ΔExpression (Log_2)	FDR *p* value*	ΔExpression (Log_2)	FDR *p* value*
		Extra cellular matrix		
Fmod	**-1.02**	**3.45E-03**	-0.02	9.96E-01
Fhdc1	**-1.03**	**6.99E-04**	-0.30	8.86E-01
Nid2	**-1.17**	**4.71E-03**	-0.44	9.13E-01
Vcan	**-1.22**	**2.65E-03**	-0.62	8.39E-01
Loxl1	**-1.23**	**2.61E-05**	-0.15	9.66E-01
Col18a1	**-1.30**	**7.91E-08**	-0.47	8.31E-01
Has3	**-1.37**	**1.66E-04**	-1.55	7.64E-02
Ltbp2	**-1.55**	**3.41E-06**	-0.67	8.80E-01
Col1a1	**-1.62**	**1.29E-08**	-0.28	8.16E-01
Lum	**-1.72**	**3.68E-02**	-0.74	8.74E-01
Optc	**-1.73**	**3.43E-08**	-0.54	9.01E-01
Fbln2	**-1.78**	**2.45E-13**	-0.41	7.71E-01
Mfap4	**-2.18**	**4.98E-13**	-0.88	8.22E-01
Col3a1	**-2.80**	**1.28E-06**	-0.58	8.83E-01

*Significant values in bold

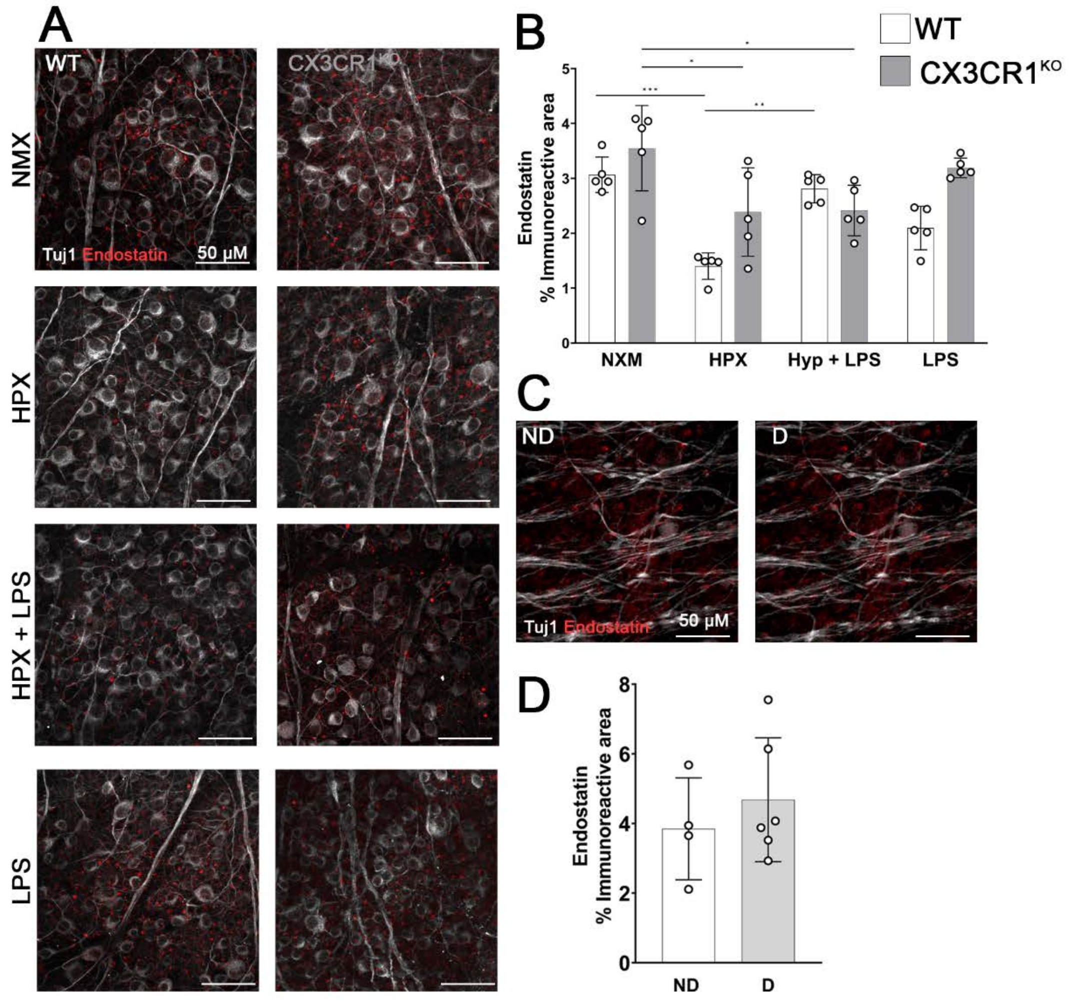

Supplemental Figure S1. Endotoxemia reverses hypoxia-induced endostatin reduction in WT but not CX3CR1^{KO} tissues. (A) Representative 40x magnification confocal images of mouse retinas stained with RGC axonal marker Tuj1 (white) and endostatin (red). (B) Quantification of endostatin immunoreactive area. Data show mean ± SD, n = 5 mice per group, dots indicate average for individual mice. (C) Representative 40x magnification confocal images of human retinas stained with RGC axonal marker Tuj1 (white) and endostatin (red). (D) Quantification of endostatin immunoreactive area. Data show mean ± SD, n = 5 mice per group, N = 4 non-diabetic human samples, n = 6 diabetic human samples. Scale bar measures 50 μm. * p <0.05, ** p <0.01, *** p <0.001, using two-way ANOVA with multiple comparisons and Tukey's correction.
